# Source attribution of carbonaceous fraction of particulate matter in the urban atmosphere based on chemical and carbon isotope composition

**DOI:** 10.1038/s41598-024-57829-x

**Published:** 2024-03-27

**Authors:** Alicja Skiba, Katarzyna Styszko, Anna Tobler, Roberto Casotto, Zbigniew Gorczyca, Przemysław Furman, Lucyna Samek, Dariusz Wideł, Mirosław Zimnoch, Anne Kasper-Giebl, Jay G. Slowik, Kaspar R. Daellenbach, Andre S. H. Prevot, Kazimierz Różański

**Affiliations:** 1 AGH University of Krakow, Faculty of Physics and Applied Computer Science, Krakow, Poland; 2 AGH University of Krakow, Faculty of Energy and Fuels, Krakow, Poland; 3 Laboratory of Atmospheric Chemistry, Paul Scherrer Institute, 5232 Villigen-PSI, Switzerland; 4 Datalystica Ltd, Park innovAARE, 5234 Villigen, Switzerland; 5 Jan Kochanowski University, Institute of Chemistry, Uniwersytecka 7 Street, 25-406 Kielce, Poland; 6 Institute for Chemical Technologies and Analytics, TU-Wien, 1060 Vienna, Austria

**Keywords:** Atmospheric chemistry, Climate-change impacts, Environmental impact

## Abstract

Air quality is of large concern in the city of Krakow, southern Poland. A comprehensive study was launched by us in which two PM fractions (PM_1_ and PM_10_) were sampled during 1-year campaign, lasting from April 21, 2018 to March 19, 2019. A suite of modern analytical methods was used to characterize the chemical composition of the collected samples. The contents of 14 sugars, sugar alcohols and anhydrosugars, 16 polycyclic aromatic hydrocarbons, selected metals and non-metals and ions were analyzed, in addition to organic and elemental carbon content. The carbon isotope composition in both analysed PM fractions, combined with an isotope-mass balance method, allowed to distinguish three main components of carbonaceous emissions in the city: (1) emissions related to combustion of hard coal, (2) emissions related to road transport, and (3) biogenic emissions. The heating season emissions from coal combustion had the biggest contribution to the reservoir of carbonaceous aerosols in the PM_10_ fraction (44%) and, together with the biogenic emission, they were the biggest contributors to the PM_1_ fraction (41% and 44%, respectively). In the non-heating season, the dominant source of carbon in PM_10_ and PM_1_ fraction were the biogenic emissions (48 and 54%, respectively).

## Introduction

The general population is increasingly aware of the dangers associated with breathing the air in which they live. It has been proven that exposure to poor quality air can lead to numerous health problems^1–7^. Krakow is the 2nd largest city in Poland, with almost 1 million inhabitants. The levels of PM_2.5_ and PM_10_ in the urban atmosphere of Krakow, in contrast to other large European cities, are regularly exceeding the air quality standards set up by the World Health Organization^8^, mainly in the autumn and winter season. To improve the air quality in the city, the Krakow City Council introduced a total ban on solid fuels combustion from September 1st, 2019^9^. The key objective from the perspective of city residents and policymakers is to identify the sources of particulate matter emissions and their contribution to the overall pollution load of the local atmosphere. There are several approaches aimed at source allocation of suspended particulate matter based on the statistical analysis of the chemical and elemental composition of the given PM fraction. Some of them have been already applied in Krakow (see e.g.^10–13^).

The carbonaceous fraction of particulate matter, besides inorganic carbon (carbonate), mostly consist of organic (OC) and elemental (EC) carbon^14^. EC is considered as a primary contaminant. It is usually emitted in incomplete combustion processes of different materials (biomass, coal, oil, petrol, etc.). Organic carbon is a mixture of different organic compounds with various functional groups incl. polycyclic aromatic hydrocarbons and saccharides (including anhydrosugars, e.g., levoglucosan, mannosan)^15,16^. The OC can be further divided into two groups, depending on its origin: (1) primary organic carbon (POC) considered as primary contaminant emitted directly to the atmosphere through the combustion of liquid and solid fuels, and (2) the secondary organic carbon (SOC), which is formed in the atmosphere through oxidation processes of organic precursors^17,18^. The OC carbon reservoir considered together with EC is called total carbon (TC). Typically, the carbonaceous fraction constitutes about 30–40% of the total urban particulate mass^19^. In previous studies conducted in Krakow in 2013 and 2017, the carbonaceous fraction constituted from 27 to 47% of PM, depending on the season of the year and the considered PM fraction^20,21^.

Particulate matter associated with local heating is represented by a wide range of elements, e.g. Cl, S, Cd, Cr, Br, As, Pb, Hg, Se—connected mostly with coal burning^22–26^. On the other hand, PM matter associated road emissions come from two main types of sources: which are (1) the exhaust processes (combustion of fuels), and (2) the non-exhaust processes (eg. abrasion of car elements like brakes and tires, resuspension of soil). The particles emitted by the first group consist of, among others, organic and elemental carbon, gases (eg. SO_2_, NO_x_), and elements like: Ni, V, Fe, Pb, Br, Al, Cd, Cu, Mg^22,27–31^. While the particles emitted by the second group are mostly linked to the presence of Zn, but also Fe, Cu, Co and Ca (when we consider tire abrasion)^27,28,31–33^. In case of the resuspension of soil we can consider several elements like: Al, Ca, K, Ce, Fe, Mg, Mn, Ti, Si, Sr, V, Co^34–36^.

The carbon isotope composition and the isotope-mass balance was already applied in Krakow to quantify the share of different carbonaceous particulate matter emission sources present in the city atmosphere^37–39^. The pilot study focusing on the total suspended particles (TSP), covering the period 2004–2010^39^, revealed large seasonal variability of carbonaceous fraction of the analysed TSP reflecting in diverse strength of different sources of carbonaceous aerosols to the local atmosphere. Two other studies were focusing on the PM_2.5_ fraction collected during the summer 2017 and winter 2018 seasons^38^ and the PM_10_ fraction collected during the summer 2018 and winter 2018/2019 seasons^37^—the results of these studies will be discussed further in Sect. 3.2.

We report here the results of carbon isotope analyses of carbonaceous fraction in the suspended particulate matter (PM_10_, PM_1_) from 1-year sampling campaign conducted in Krakow from April 2018 to March 2019. Both ^14^C/^12^C and ^13^C/^12^C isotope ratios were quantified in the collected PM_10_ and PM_1_ samples. The results of carbon isotope analyses, combined with isotope-mass balance approach, were used to quantify the contribution of three sources of carbon in the analyzed PM_10_ and PM_1_ samples and their seasonal variability. The isotope analyses were complemented by concentration measurements of ions (cations and anions), selected elements, as well as polycyclic aromatic hydrocarbons (PAHs) and carbohydrates such as sugar alcohols and anhydrosugars which are part of the carbon reservoir in the analyzed PM fractions. Anhydrosugars are formed as a result of pyrolysis of materials containing cellulose or hemicellulose^40–43^. The most important among them is levoglucosan, which is a recognized marker of biomass combustion^44,45^.

So far, such a long (approx. 1 year) measurement campaign covering two PM fractions (PM_1_ and PM_10_) has not been carried out in Krakow. It is worth to notice that such comprehensive analysis of the PM_1_ fraction, which is so far less characterized and studied PM fraction, than the fraction of larger diameter, has never been conducted in Krakow. Additionally, as far as authors know, PM_1_ was never used in Poland before, to provide isotope mass-balance. The timing of the research is also unique (just before the introduction of the above-mentioned ban on solid fuels combustion in the city). Hence, this work is the last such in-depth characterization of particulate matter and source attribution of its carbonaceous fraction before this period.

## Sampling and methods

### Sampling campaign

**Figure 1:**
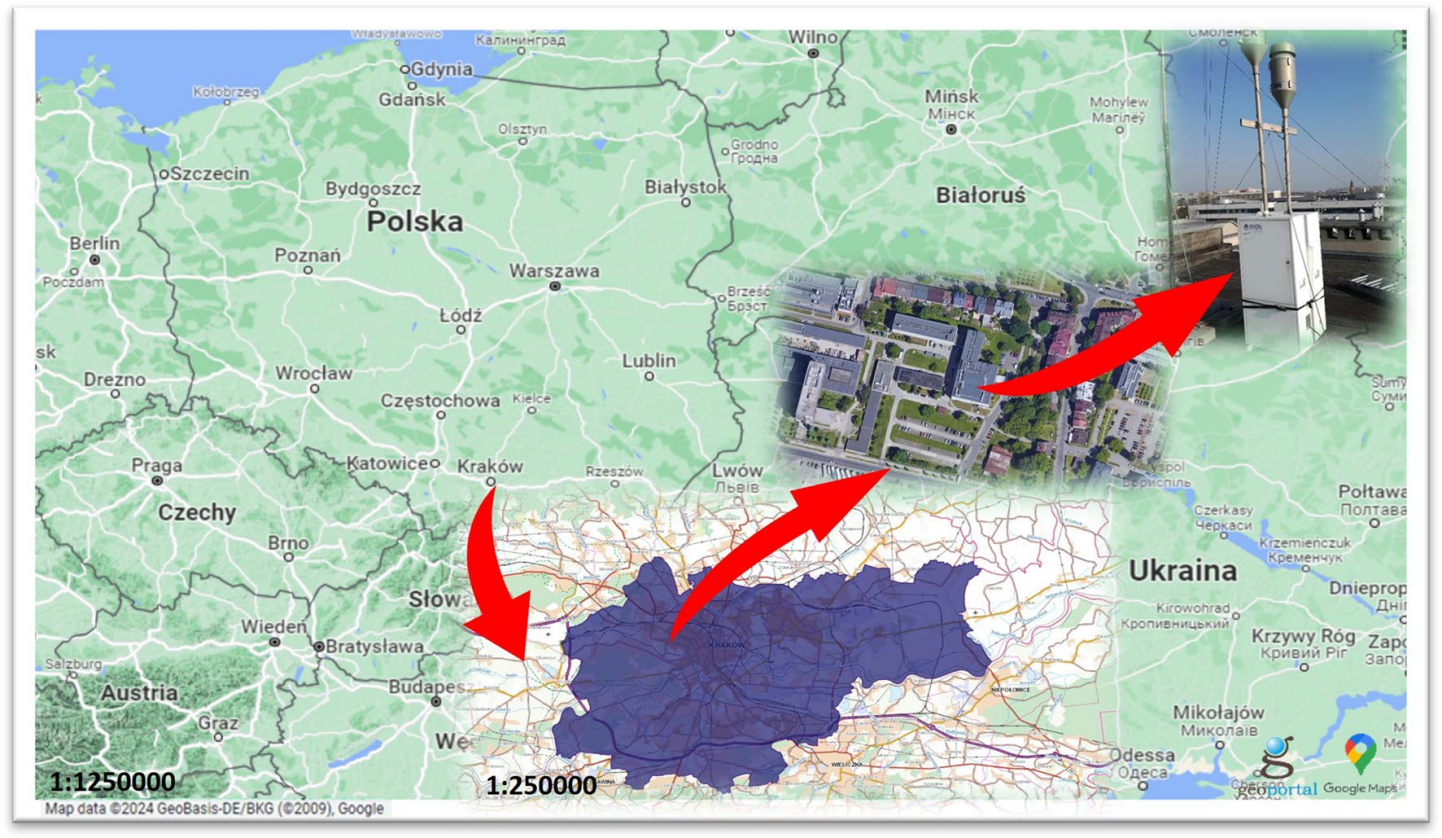
Sampling site. The red arrows inside the figure indicate the exact location of the high-volume samplers in each picture included (sources: www.google.com/maps, www.geoportal.gov.pl, authors’ photographs, the picture was edited using MS Office tools).

Daily samples of two particulate matter fractions (PM_1_ and PM_10_) were collected from April 21, 2018 to March 19, 2019, except for the period from September 28, till October 25, 2018. Two high-volume samplers (DHA-80, Digitel, Elektronik AG, Hegnau, Switzerland, air flow 720 m^3^ day^−1^) were placed on the rooftop of the Faculty of Physics and Applied Computer Science building, AGH University of Krakow (50° 04ʹ N 19° 55ʹ E, 220 m a.s.l.) at the height of about 20 m above the local ground (Fig. 1). Daily samples of PM_1_ and PM_10_ were collected on Pallflex® quartz filters (diameter of 15 cm). Sampling was carried out according to the procedure which was further explained in the “Supplementary Information” (section A). From the collected material, every fourth sample was further analysed.

### Carbonaceous fraction measurements

Organic and elemental carbon concentration (OC, EC) was analysed in the circular punches of individual filters (ø 10 mm) by thermal-optical method using Sunset Laboratory OCEC Lab Aerosol Analyzer (USA) using the EUSAAR2 (European Supersites for Atmospheric Aerosol Research) protocol^46^. More information about the method is presented in the Supplementary Information.

### Carbon isotope analyses and isotope-mass balance approach

The analysed filters were aggregated into sets representing approximately monthly periods, to obtain a sufficient amount of carbon for isotope analyses (> 1 mg). The obtained results were then assigned to the heating or non-heating season. Aggregation periods and the corresponding weighted averages of the total carbon present in the aggregated samples representing both fractions are presented in Table 1S (Supplementary Information). The total carbon (TC) reservoir in the analysed aggregated samples was transformed into gaseous form (CO_2_) which was later subjected to cryogenic purification. The method is presented in detail in^38,47^. The ^13^C/^12^C ratios of the obtained carbon dioxide were quantified using isotope ratio mass spectrometry (IRMS). The radiocarbon content was measured with accelerator mass spectrometry (AMS) on the 1.5 SDH-Pelletron Model Compact Carbon AMS^48^. The accelerator mass spectrometry analyses were done after the IRMS analysis (CO_2_ was recovered after isotope ratio mass spectrometry).

The measured ^13^C/^12^C ratios are expressed as δ values defined as per mille deviations from the internationally accepted reference standard^49,50^, whereas the radiocarbon contents in the analysed samples are expressed as percent of modern carbon (pMC) defined as^51–53^:

1 $$pMC=\frac{{}{}^{14}R{ }_{SN}}{{}{}^{14}R{ }_{ON}}\times {10}^{2}$$

where ^*14*^*R *_*SN*_ and ^*14*^*R *_*ON*_ are ^14^C/^12^C isotope ratios measured in the analysed samples and the reference standard (Oxalic Acid II), respectively. The measured isotope ratios were then used to calculate the contribution of pre-defined emission sources of carbon using isotope-mass balance approach^38^:

2 $$1= {F}_{bio}+{F}_{coal}+{F}_{traff}$$

3 $${\delta }^{13}{C}_{TC}={\delta }^{13}{C}_{bio} \times {F}_{bio}+ {\delta }^{13}{C}_{coal} \times {F}_{coal}+ {\delta }^{13}{C}_{traff} \times {F}_{traff}$$

4 $${pMC}_{TC}={pMC}_{bio}\times {F}_{bio}+{pMC}_{coal}\times {F}_{coal}+{pMC}_{traff}\times {F}_{traff}$$

where: *F*_*bio*_*,F*_*coal,*_* F*_*traff*_ stand for mass fractions of carbon originating from biogenic emissions, coal combustion and road transport emissions, respectively. *δ*^*13*^*C*_*TC*_ is the ^13^C isotope composition of the measured PM_1_ or PM_10_ samples. *δ*^*13*^*C*_*bio, coal, traff*_ stands for the assigned ^13^C isotope signatures of biogenic emissions, coal combustion and road transport emissions, respectively. *pMC*_*TC*_ signifies percent of Modern Carbon of the analysed PM_1_ or PM_10_ samples, whereas *pMC*_*bio, coal, traff*_ stands for assigned percent of modern carbon values for biogenic emissions, coal combustion and road transport emissions, respectively. The mass fractions (*F*_*bio*_*, F*_*coal,*_* F*_*traff*_) of the total carbon reservoir present in the monthly composite samples of PM_1_ and PM_10_ can be calculated from Eqs. (2–4) above, provided that all other variables are either measured or derived from other sources.

A first step in calculating the contributions of the pre-defined emission sources of carbon (biogenic emissions, coal combustion and road transport) to the overall carbon budget of the analysed PM fractions of particulate matter is the appropriate assessment of ^13^C and ^14^C isotope signatures of those sources. For example, the δ^13^C_coal_ values related to coal combustion emission can differ significantly on a global scale, depending on the geographical origin and age of the burned coal (from − 30.1 to − 23.3‰, as reported in Ref.^54^). The δ^13^C_coal_ values of hard coal mined in Poland were thoroughly characterized. They are in a relatively narrow range, from − 24.5 to − 23.3‰^55^. The δ^13^C_coal_ value of − 23.3‰ was chosen for this work. The δ^13^C_traff_ values reported for road transport emissions have the range between − 28.3 and − 24.5‰^38,54^. For this study, the average δ^13^C_traff_ value equal − 27.6‰, derived from the data reported in^54^ was adopted. This value is comparable with the results obtained by the authors in road tunnel in Krakow, where this signature was in the range from − 27.1 to − 27.8‰, depending on the PM fraction analysed^56^. The emissions of carbon associated with the road transport are mainly related to combustion of liquid fuels, but also to the wear of car tires and asphalt pavement.

If only petroleum would be considered, which is a fossil fuel devoid of radiocarbon, the *pMC*_*traff*_ value of the particles associated with combustion of such fuel would be zero, as is the case with hard coal combustion (*pMC*_*coal*_). However, considering additional factors included in road emissions, such as the use of natural rubber in the automotive industry to produce car tires (approx. 15–20%), as well as the use of biofuels (the regulations in force in Poland and the European Union state that at least 10% of the petrol must be a biocomponent, for diesel fuel it is 7%^57^), it was assumed that ^14^C content in the carbon emissions from road transport is constant and equal 10 pMC^23^.

The ‘biogenic emissions’ category includes pollen/plant spores (emitted mainly during the growing season) and secondary organic aerosol (SOA), as well as wood and biomass combustion, which is mostly present during colder months of a year. According to previous studies^38^, the δ^13^C_bio_ values range in Krakow from − 28 to − 24‰. Constant δ^13^C_bio_ value equal − 25‰ was adopted in this work. Due to the fact that pollen and plant spores, as well as volatile organic compounds (VOCs) emitted by living plants should have the ^14^C contents resembling those in the local atmosphere (after accounting for isotope fractionation steps involved in the photosynthesis process), we adopted in the isotope-mass balance calculations the constant *pMC*_*bio*_ value equal 105% for non-heating season. This choice was guided by the fact that radiocarbon levels in the local atmosphere (atmospheric CO_2_) fluctuate nowadays between 100 and 117% (unpublished data). For heating season, a slightly higher *pMC* value equal 115% was adopted due to the fact that burning of wood is the major source of carbonaceous aerosols in the city atmosphere during this time period of a year and the mean age of wood (time elapsed from its time of formation) is in the order of 10–15 years. At that time period the *pMC* levels in the city atmosphere were in the order of 115–120%^58^.

Due to the fact that carbon is present also in the mineral fraction of the analysed PM_1_ and PM_10_ fractions of suspended particulate matter, appropriate corrections of the measured *δ*^*13*^*C*_*TC*_ and *pMC*_*TC*_ values need to be applied to account for the presence of calcium carbonate. Details of the correction procedure are presented in^39^^.^ The corrections derived for 19 analysed samples of PM_1_ and PM_10_ fractions resulted in 0.3% and 0.2‰ for *pMC*_*TC*_ and *δ*^*13*^*C*_*TC*_, respectively. As they are comparable with the measurement uncertainties of isotope analyses (ca. 0.2–0.3% for AMS and 0.1–0.2‰ for IRMS), no adjustment of the measured *δ*^*13*^*C*_*TC*_ and *pMC*_*TC*_ values was made.

### Chemical analysis

Inorganic anions (F^−^, Cl^−^, NO_2_^−^, Br^−^, NO_3_^−^, PO_4_^3−^, SO_4_^2−^) and cations (Na^+^, K^+^, Mg^2+^, Ca^2+^, NH_4_^+^) concentrations were analysed with isocratic ion chromatography (IC) performed with ICS-1100 instrument (Thermo Scientific), equipped with Thermo Scientific (Dionex) AS-DV autosampler and ion-exchange columns. The details of the method are presented in the Supplementary Information.

Sixteen polycyclic aromatic hydrocarbons (acenaphthene (Acn), acenaphthylene (Acy), anthracene (Ant), benzo[b]fluoranthene (BbF), benzo[a]anthracene (BaA), benzo[a]pyrene (BaP), benzo[ghi]perylene (BghiP), benzo[k]fluoranthene (BkF), chrysene (Chry), dibenzo[ah]anthracene (DahA), fluoranthene (Flt), fluorene (Flu), indeno[1,2,3-cd]pyrene (IP), naphthalene (Nap), phenanthrene (Phen) and pyrene (Pyr) were analysed with gas chromatography coupled with mass spectrometry system (GC/MS). Model Clarus 600/600T from Perkin Elmer (USA) was used. Polycyclic aromatic hydrocarbons were separated on the Elite-5MS (30 m × 0.25 mm × 0.25 µm) capillary column. Altogether 48 aggregated samples (2, 3 or 4 days—see Table 2S, Supplementary Information) were analysed for PM_1_ and PM_10_ fractions (24 samples for each fraction). The details of the procedure are described in^59^, the method was slightly modified in order to analyse polycyclic aromatic hydrocarbons. The retention times, characteristic ions of tested analytes and validation parameters of the method are shown in Table 3S (Supplementary Information).

The high-performance anion-exchange chromatography with pulsed amperometric detection (HPAE-PAD) was used to analyse 14 sugars, sugar alcohols and anhydrosugars including: inositol, erythritol, xylitol, levoglucosan, arabitol, mannosan, trehalose, mannitol, galactosan, glucose, fructose, galactose, cellobiose and sucrose. Altogether 81 samples (with a diameter of 10 mm) of each fraction (PM_1_ and PM_10_) were extracted in 3 ml of Milli-Q water, ultrasonicated for 30 min, and centrifuged at 4000 rpm for 10 min. The extracts were analyzed with a Dionex™ ICS3000 (Thermo Scientific™), equipped with a CarboPac™ MA1 column, utilizing a sodium hydroxide gradient of 480–650 mM and a flow rate of 0.4 ml·min^−1^. The method is described in detail in Ref.^60^. The limit of detection (LOD) was determined as the minimum concentration that was visible on the chromatogram and produced a peak height at least three times the signal to noise ratio. The LOD values were in the range of 0.004–0.05 µg·m^−3^.

The elemental analysis of the filter material comprised 17 elements: Cl, K, Ca, Ti, V, Cr, Mn, Fe, Co, Ni, Zn, Cu, Br, Rb, Sr, As and Pb. The concentrations of the measured elements were quantified by the energy dispersive X-ray fluorescence (EDXRF) method. Details of elemental analysis are presented in the Supplementary Information. Detection limits and detailed description of the method is presented elsewhere^12,20^.

### Chemical mass closure method

The mass closure method is a semi-quantitative method based on comparison of the gravimetrically determined mass concentration of a TSP (or each fraction of PM), with the sum of the measured concentrations of individual components of TSP or chosen PM fraction, respectively^61^. The mass closure method is useful to perform preliminary qualitative analysis of possible emission sources of particulate matter based on temporal and spatial variability of chemical composition of the analysed PM fraction^10,61–69^. The reconstructed chemical composition of particulate matter usually consists of about seven representative chemical components. Frequently considered components are as follows: the secondary inorganic ions, elemental carbon, organic matter, crustal matter (also referred to as soil dust), salt (road salt or sea salt, depending on the location), trace elements and the last group generally referred to as not-identified matter (other, etc.). The emission categories adopted in this work are presented in Table 4S (Supplementary Information).

## Results and discussion

### Carbonaceous fraction characteristics, concentrations of polycyclic aromatic hydrocarbons and carbohydrates

The PM samples collected in the framework of this study were characterized by a high content of organic carbon in total carbon reservoir (from 75 to 88%, depending on the considered PM fraction and the month, as determined by the thermal-optical method). PM weight average during each month of the campaign is presented in Table 1. For PM_1_ it was in the range of 17–43.1 µg·m^−3^ and for PM_10_ in the range of 38.3–107.4 µg·m^−3^, respectively. As expected, higher PM averages were noticed during months representing heating season in Krakow.

**Table 1:** Monthly average results for PAHs and sugars identified in PM_10_ and PM_1_ followed by PM weight average and percentage share of carbon from PAHs and sugars in the organic carbon reservoir.

Name	Date (every 4th sample)	PM_10_									PM_1_								
		PM_10_ weight average [µg m^−3^]	Determined sugars	Total no. of determined sugars	Share of carbon from sugars in OC [%]	Determined PAHs	Total no. of determined PAHs	Share of carbon from PAHs in OC [%]	OC avg. [µg·m^−3^]	TC avg. [µg·m^−3^]	PM_1_ weight average [µg·m^−3^]	Determined sugars	Total no. of determined sugars	Share of carbon from sugars in OC [%]	Determined PAHs	Total no. of determined PAHs	Share of carbon from PAHs in OC [%]	OC avg. [µg·m^−3^]	TC avg. [µg·m^−3^]
April_2018	21.04–29.04.2018	69.5	**levoglucosan arabitol, mannosan, glucose**	4	3.1	**Phen, Flt, Pyr, BaA, Chry, BbF, BkF, BaP, IP, BghiP**	10	0.008	6.7	8.1	22.8	**Levoglucosan**	1	1.3	**BbF**	1	0.0007	3.6	4.5
May_2018	03.05–31.05.2018	60.1	**levoglucosan, arabitol, glucose**	3	1.2	**Phen, Flt, Pyr, BaA, Chry, BbF, BkF, BaP, IP, BghiP**	10	0.005	5.6	6.8	17.4	**Levoglucosan**	1	0.7	**BbF**	1	0.001	2.9	3.6
June_2018	04.06–28.06.2018	38.3	**levoglucosan, arabitol, glucose**	3	1.1	**Phen, Flt, Pyr, BaA, Chry, BbF, BkF, BaP**	8	0.007	5.6	6.7	17	**Levoglucosan**	1	0.5	**BbF**	1	0.0008	3.2	3.9
July_2018	02.07–30.07.2018	48.4	**levoglucosan arabitol, mannitol, glucose**	4	2.3	**Phen, Flt, Pyr, BaA, Chry, BbF, BkF, BaP, IP, BghiP**	10	0.008	5.4	6.4	18.2	**Levoglucosan**	1	0.8	**Flt, Pyr, BbF**	3	0.001	3.2	3.6
August_2018	03.08–31.08.2018	48	**levoglucosan arabitol, glucose**	3	1.5	**Phen, Flt, Pyr, BaA, Chry, BbF, BkF, BaP, IP, BghiP**	10	0.01	5.4	7.0	19.7	**Levoglucosan, glucose**	2	1.1	**Flt, Pyr, BbF**	3	0.001	3.2	4.0
September_2018	04.09–27.09.2018	65.6	**levoglucosan arabitol, mannosan, galactosan, glucose**	5	2.4	**Phen, Flt, Pyr, BaA, Chry, BbF, BkF, BaP, IP, BghiP**	10	0.004	6.0	7.4	22.7	**Levoglucosan**	1	1.2	**Flt, BbF**	2	0.002	4.3	5.5
October_2018	26.10–30.10.2028	41.2	**levoglucosan arabitol, mannosan, galactosan, glucose**	5	2.7	**Phen, Flt, Pyr, BaA, Chry, BbF, BkF, BaP, IP, BghiP**	10	0.01	5.9	7.2	24.8	**Levoglucosan**	1	0.5	**Phen, Flt, Pyr, BaA, Chry, BbF, BkF, BaP, IP, BghiP**	10	0.04	2.9	3.8
November_2018	03.11–27.11.2018	91.7	**inositol, levoglucosan arabitol, mannosan, trehalose, galactosan, glucose**	7	3.9	**Phen, Ant, Flt, Pyr, BaA, Chry, BbF, BkF, BaP, IP, DahA, BghiP**	12	0.06	16.0	18.9	43.7	**Inositol, levoglucosan, mannosan**	3	3.0	**Phen, Flt, Pyr, BaA, Chry, BbF, BkF, BaP, IP, DahA, BghiP**	11	0.06	7.0	9.0
December_2018	01.12–29.12.2018	86.6	**inositol, levoglucosan arabitol, mannosan, trehalose, galactosan, glucose**	7	2.7	**Nap, Acy, Flu, Phen, Ant, Flt, Pyr, BaA, Chry, BbF, BkF, BaP, IP, DahA, BghiP**	15	0.08	18.6	21.0	41.2	**Inositol, levoglucosan, mannosan, galactosan**	4	2.8	**Nap, Acy, Phen, Ant, Flt, Pyr, BaA, Chry, BbF, BkF, BaP, IP, DahA, BghiP**	14	0.08	7.7	9.3
January_2019	02.01–30.01.2019	82	**inositol, levoglucosan arabitol, mannosan, trehalose, galactosan, glucose, cellobiose**	8	3.0	**Acy, Flu, Phen, Ant, Flt, Pyr, BaA, Chry, BbF, BkF, BaP, IP, DahA, BghiP**	14	0.08	14.4	16.4	26.6	**Levoglucosan, mannosan**	2	1.7	**Nap, Phen, Ant, Flt, Pyr, BaA, Chry, BbF, BkF, BaP, IP, DahA, BghiP**	13	0.05	6.1	7.0
February_2019	03.02–27.02.2019	107.5	**inositol, levoglucosan arabitol, mannosan, galactosan, glucose**	6	2.2	**Nap, Acy, Flu, Phen, Ant, Flt, Pyr, BaA, Chry, BbF, BkF, BaP, IP, DahA, BghiP**	15	0.15	19.8	23.4	40	**Levoglucosan, mannosan, galactosan**	3	2.5	**Phen, Ant, Flt, Pyr, BaA, Chry, BbF, BkF, BaP, IP, DahA, BghiP**	12	0.08	7.0	8.7
March_2019	03.03–15.03.2019	63.1	**levoglucosan mannosan, galactosan, glucose**	4	2.1	**Nap, Acy, Phen, Ant, Flt, Pyr, BaA, Chry, BbF, BkF, BaP, IP, DahA, BghiP**	14	0.09	7.7	9.2	30.8	**Inositol, levoglucosan**	2	2.0	**Phen, Flt, Pyr, BaA, Chry, BbF, BkF, BaP, IP, BghiP**	10	0.04	3.5	4.2

The analysed sugars and PAHs constituted from 0.5 to 4% of the OC concentration depending on the month. The highest share (4%) was determined in November 2018 for the PM_10_ fraction, while the lowest (0.5%) in October 2018 for the PM_1_ fraction. In the same month (October 2018) the ratio OC/TC was also the lowest in the entire measurement period (75%). Depending on the month, from 1 to 4 saccharides and from 1 to 14 PAHs were identified in the PM_1_ fraction, to be compared with 3–8 saccharides and from 8 to 15 PAHs for PM_10_ fraction (cf. Table 1). The largest number of individual types of organic compounds identified in both fractions was for the period from November 2018 to February 2019, i.e., during the heating season in Krakow.

**Figure 2:**
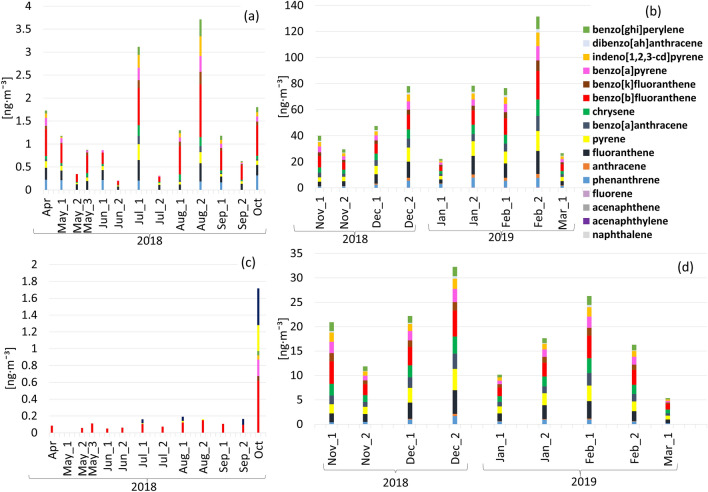
(**a**–**d**) Seasonal variability of PAHs concentrations in PM_10_ (**a**, **b**) and PM_1_ (**c**, **d**) fractions during the study period.

**Figure 3:**
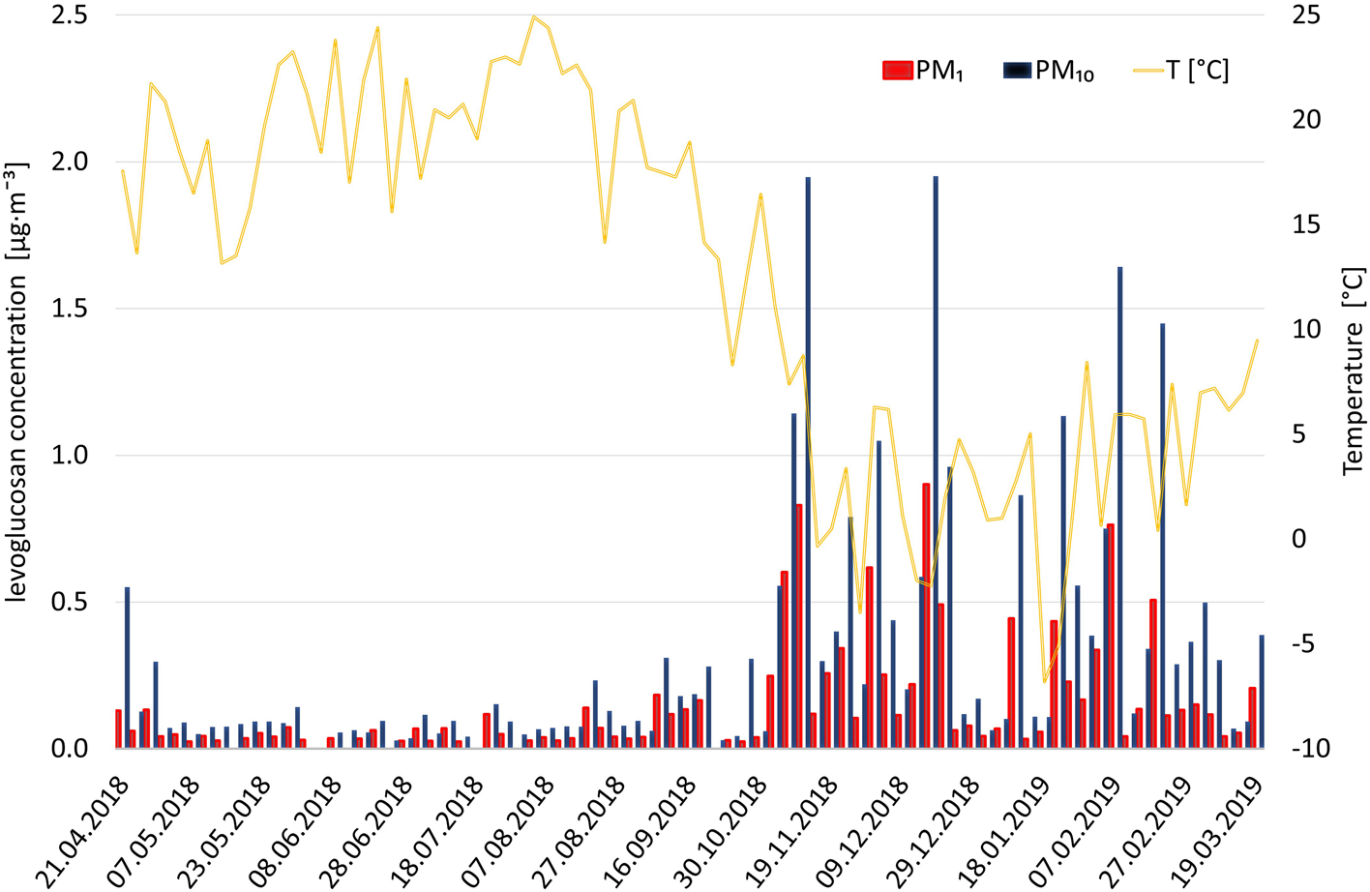
Seasonal variability of levoglucosan concentrations in PM_10_ and PM_1_ fractions, with ambient air temperature during the study period shown in the background.

The results of carbohydrates and polycyclic aromatic hydrocarbons analyses are summarized in Tables 5S and 6S (Supplementary Information). The PAHs results are presented in Fig. 2a–d. Levoglucosan is presented in Fig. 3 as the only carbohydrate detected in the vast majority of samples (74 out of 81 for the PM_1_ fraction and 78 out of 81 for the PM_10_ fraction) among the 14 analysed. As seen in Figs. 2 and 3, the concentrations of all measured PAHs and levoglucosan are higher during the heating season in Krakow (autumn and winter), which is characterized by intensified combustion of coal and wood for heating purposes. Among sixteen analysed PAHs, the highest maximum concentrations in aggregated samples were recorded for Fluoranthene, Pyrene and Benzo[b]fluoranthene (17.64 ng m^−3^, 15.31 ng m^−3^, 21.94 ng m^−3^) in PM_10_ (aggregated dates: 19.02, 23.02 and 27.02.2019) and (4.85 ng m^−3^, 4.38 ng m^−3^, 5.35 ng m^−3^) in PM_1_ (aggregated dates: 17.12, 21.12, 25.12 and 29.12.2018). The maximum concentration of levoglucosan was 0.9 μg m^−3^ (in PM_1_ fraction) and 1.95 μg m^−3^ (in PM_10_ fraction), both values measured on December 17, 2018.

The observed average concentrations of PAHs in the PM_10_ fraction are approximately 4 times lower than those observed in Krakow in winter 2014^70^. This may indicate an improvement in air quality over a period of ca. 5 years in Krakow. PAHs average concentrations determined in this study are usually lower than those recorded in other European cities in previous years (the exception is the result from Athens)^71–73^. The results from this study for PM_1_ fraction are similar to those obtained in Brazil^74^. This study was similar in terms of the duration. Table 6S (Supplementary Information) shows the comparison of average concentrations of PAHs obtained in this study and those reported by other research teams for different areas around the world.

### Source attribution of carbonaceous aerosols based on isotope-mass balance approach

The numerical results of *δ*^*13*^*C*_*TC*_ and *pMC*_*TC*_ analyses of the aggregated monthly samples and the data used in isotope-mass balance equations are shown in Fig. 1S and presented in Table 7S, respectively (Supplementary Information).

**Figure 4:**
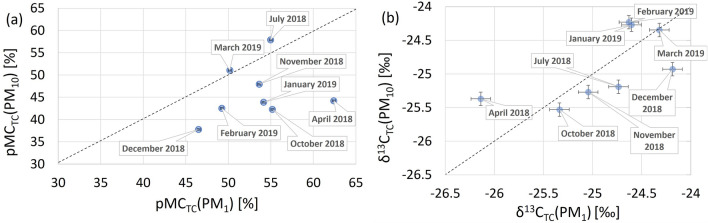
(**a**, **b**). Relationships between radiocarbon contents expressed in pMC (**a**) and δ^13^C_TC_ values (**b**) measured in aggregated monthly PM_1_ and PM_10_ samples. Measurement uncertainties of the data points in (**a**) are of the size of symbols used.

The measured *δ*^*13*^*C*_*TC*_ values ranged from − 26.1‰ (April 2018) to − 24.2‰ (December 2018) for the PM_1_ fraction and from − 25.5‰ (October 2018) to − 24.2‰ (February 2019) for the PM_10_ fraction. Higher δ^13^C_TC_ values were recorded in both fractions in the period from December 2018 to March 2019. Lower values of δ^13^C_TC_ indicate the dominance of biogenic sources while higher values indicate enhanced contribution of hard coal combustion. The measured radiocarbon content in the aggregated samples, expressed in pMC, ranged from 37.8% (December 2018) to 57.9% (July 2018) for the PM_10_ fraction, and for PM_1_ from 46.5% (December 2018) to 62.4% (April 2018). As expected, lower levels of radiocarbon were recorded in cooler months, reflecting enhanced emissions of carbonaceous aerosols devoid of radiocarbon, associated with combustion of coal in the city for heating purposes. Figure 4 shows scatter plots illustrating mutual relations between the isotope parameters (*δ*^*13*^*C*_*TC*_ and *pMC*_*TC*_) measured in both analysed PM fractions, for the 8 months for which both isotope parameters were measured (see list in Table 1S in Supplementary Information).

As seen in Fig. 4a, the data points cluster below the 1:1 line, indicating generally higher level of radiocarbon content in PM_1_ fraction when compared to the PM_10_ fraction which can be linked to higher contribution of biogenic carbonaceous aerosols characterized by high pMC values in the fine fraction (PM_1_), or higher contribution of carbonaceous aerosols associated with burning of fossil fuels devoid of radiocarbon, accumulating in the PM_10_ fraction. On the other hand, in Fig. 4b δ^13^C_TC_ values do not provide clear seasonal information, compared to the pMC results.

**Figure 5:**
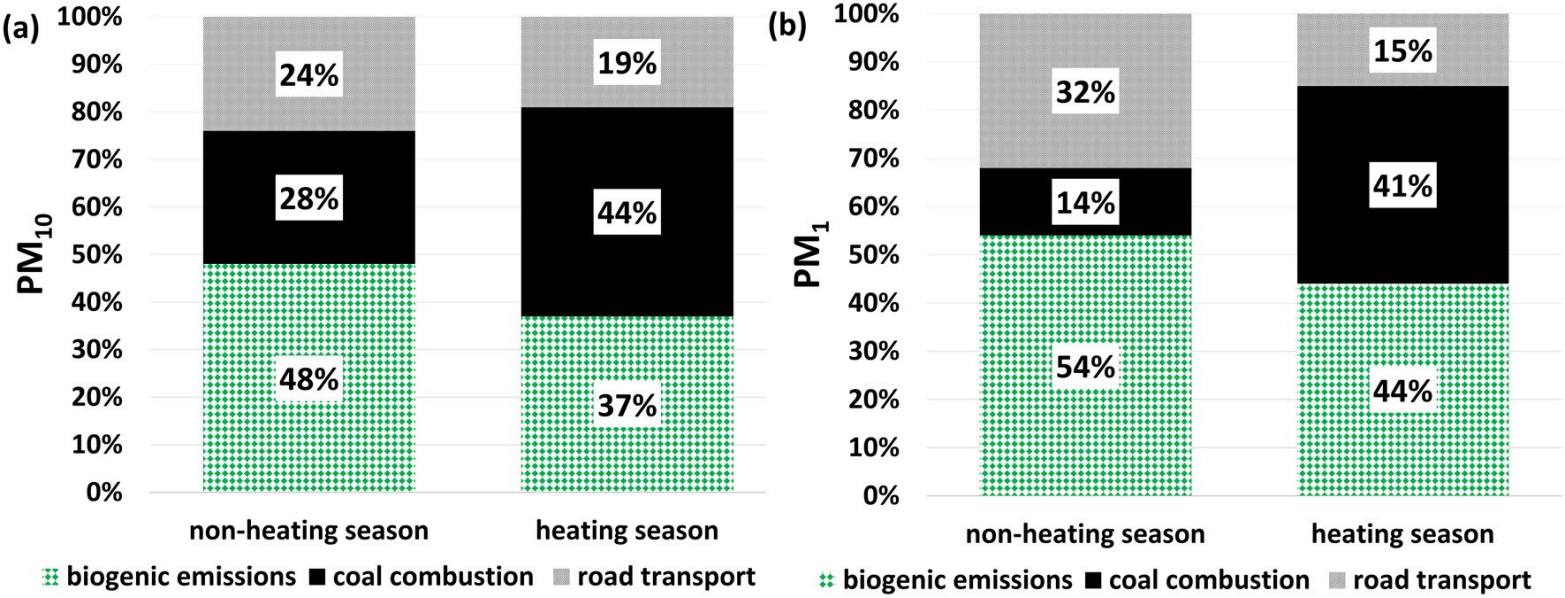
(**a**, **b**). Seasonal variability of the mass fractions of carbon originating from biogenic emissions, from coal combustion and emissions related to road transport (F_bio_, F_coal_, F_traff_, respectively), present in the carbonaceous fraction of the analysed PM_10_ (**a**) and PM_1_ (**b**) samples collected in Krakow in the period April 21, 2018–March 19, 2019, derived from the isotope-mass balance approach (Eqs. 2–4).

The set of isotope-mass balance equations (Eqs. 2–4) was solved for *F*_*bio*_*, F*_*coal,*_* F*_*traff*_ mass fractions, separately for heating and non-heating season and for both PM fractions. Heating season contained 6 aggregated monthly samples (for both fractions of PM), while non-heating season 3—in case of PM_10_ and 4—in case of PM_1_ (see list in Table 1S in Supplementary Information). The results are presented in Fig. 5a,b. During the non-heating season, the biogenic emissions were a dominant carbon source in the PM_1_ fraction with a share of 54%. These emissions are related to the combustion of biomass, as well as to emissions from the biosphere (emissions of pollen, spores and VOCs and subsequent oxidation during the vegetation period). The results of the analyses of the sources of organic aerosols contained in the PM_1_ fraction, carried out in Krakow with the use of the Q-ACSM (quadrupole aerosol chemical speciation monitor), showed that the presence of sources related to biomass combustion in the summer can reach up to 11% ^13^. This would point to a dominant role of living biosphere in the biogenic emissions of carbon during spring and summer season. Emissions of carbon associated with road transport were second in the importance (approx. 32%). The lowest share was attributed to emissions related to coal combustion (ca. 14%). Partitioning of the emission sources of carbon present in the PM_1_ fraction varies substantially during the heating season (autumn and winter). The biogenic emissions, which are mostly associated during this period with biomass combustion, decrease to about 44%. The share of emissions related to coal combustion increases to approximately 41% during the heating season, while the share of emissions associated with the road transportation drops to ca. 15%. However, the lower emission shares obtained for road transportation in the colder seasons do not indicate a reduction car traffic in Krakow in autumn and winter. Rather, they result from an increase in the carbon reservoir in the PM_1_ fraction associated with dominant emissions from the biomass and coal combustion for heating purposes in the city during this period (average concentration of PM_1_ increases from 11.9 to 19.0 μg m^−3^, to be compared with an increase from 34.0 to 47.2 μg m^−3^ for PM_10_ fraction).

Similar relations between emission sources are observed for the PM_10_ fraction of the suspended particulate matter. During the heating season, the mass fractions of carbon derived from the isotope-mass balance approach amounts to 44% (coal combustion), 37% (biogenic emissions) and 19% (road transport). For the non-heating season the biogenic emissions dominate, with approx. 48%, while the share of carbon emissions associated with road transportation is around 24%. However, the calculated mass fractions of combustion of coal during the non-heating season appear unexpectedly high (approximately 28%), when compared to the PM_10_ fraction in similar studies carried out in Krakow (cf. Table 2) (where the contribution of the coal combustion during summer ranges from 5 to 10%), and to the PM_1_ fraction in this study (14%). Thus, the results from different studies are comparable to the results of PM_1_^37,38^. However, the comparison between PM_10_ collected during winter 2018/2019 season in urban background site and presented in^37^, as well as PM_10_ from this study during heating-season 2018/2019 shows very similar values (discrepancy of up to 2%) (Table 2).

**Table 2:** Comparison of the percentage shares of emission sources obtained in this work with the shares of the same sources obtained in other studies for the atmosphere of Krakow.

Source	Summer 2018^a^		Winter 2018/2019^a^		Summer 2017^b^	Winter 2018^b^	Non-heating season 2018^c^		Heating season 2018/2019^c^	
	Traffic	Urban	Traffic	Urban	Urban	Urban	Urban	Urban	Urban	Urban
	PM_10_	PM_10_	PM_10_	PM_10_	PM_2.5_	PM_2.5_	PM_10_	PM_1_	PM_10_	PM_1_
Biogenic	33	47	32	39	52.5	37.1	48	54	37	44
Coal	10	5	35	42	7.2	54.3	28	14	44	41
Traffic/road transport	57	48	33	19	40.3	8.6	24	32	19	15

^a^Samek et al.^37^.

^b^Zimnoch et al.^38^.

^c^This work.

To sum up, the above-presented results show that during the heating season emissions from coal combustion had the biggest contribution to the PM_10_ carbonaceous aerosol reservoir (44%), and together with the biogenic emissions they were the biggest contributors to PM_1_ (41% and 44%, respectively). In the non-heating season, the dominant source of carbon were biogenic emissions (48% of PM_10_, 54% of PM_1_).

Table 3 shows the comparison of the signatures used in the previous studies conducted in Krakow and the signatures used for this work. We can see that most of the signatures are comparable to each other (at least in terms of the assumed uncertainties), the only exception is δ^13^C_traff_, where the value assumed in previous studies (− 30 ± 1‰) differs from that adopted for this publication (− 27.6‰), which might indicate one of the reasons for the differences between the results in previous researches conducted in Krakow and the results obtained in this work.

**Table 3:** Comparison of values of the signatures used in the isotope-mass balance in different studies conducted in Krakow.

Signature	Unit	Samek et al.^37^*	Zimnoch et al.^38^	This work	
				Non-heating	Heating
δ^13^C_bio_	[‰]	(− 24.0 ± 1.5)	(− 26 ± 2)	− 25	− 25
δ^13^C_coal_	[‰]	(− 23.3 to − 24.5) range**	(− 23.3 to − 24.5) range**	− 23.3	− 23.3
δ^13^C_traff_	[‰]	(− 30 ± 1)	(− 30 ± 1)	− 27.6	− 27.6
pMC_bio_	[%]	110 ± 5	110 ± 5	105	115
pMC_traff_	[%]	no data	10 ± 1	10	10
pMC_coal_	[%]	0	0	0	0

*The study does not provide isotopic signatures, there is a reference to the publications^38,39^, so to not duplicate the results from^38^, we present^39^.

**No exact value in the provided range is given.

To assess the uncertainties associated with the contribution of three emission sources discussed above, the sensitivity analysis was used (see Fig. 2S, Supplementary Information). The maximum influence parameter was defined which is the ratio of the calculated change of the given component of the emissions derived from the isotope mass balance to the assumed range of the given parameters (pMC and ^13^C value). The calculated biogenic component of the emissions was the most sensitive function of the assumed radiocarbon signature of this component. The maximum influence parameter was equal 0.4% per 1 pMC. The road transport component of the emissions appeared to be less sensitive to the assumed pMC signature of this component (Fig. 2S Supplementary Information).

For the ^13^C isotope composition, the assumed isotopic signature of the coal combustion source in heating season has the highest influence on the calculated coal combustion contribution reaching up to 14.9% per 1‰, this translates to ca. 3% uncertainty of the derived component of the balance (assuming the measurement uncertainty of δ^13^C values 0.2‰). In conclusion, the sensitivity analysis showed that the uncertainty of the estimated carbon balance components for the analysed period is in the order of a few percent. Details of the method are presented in^38^.

### ***Attribution of PM***_***1***_*** and PM***_***10***_*** sources based on mass closure method***

**Figure 6:**
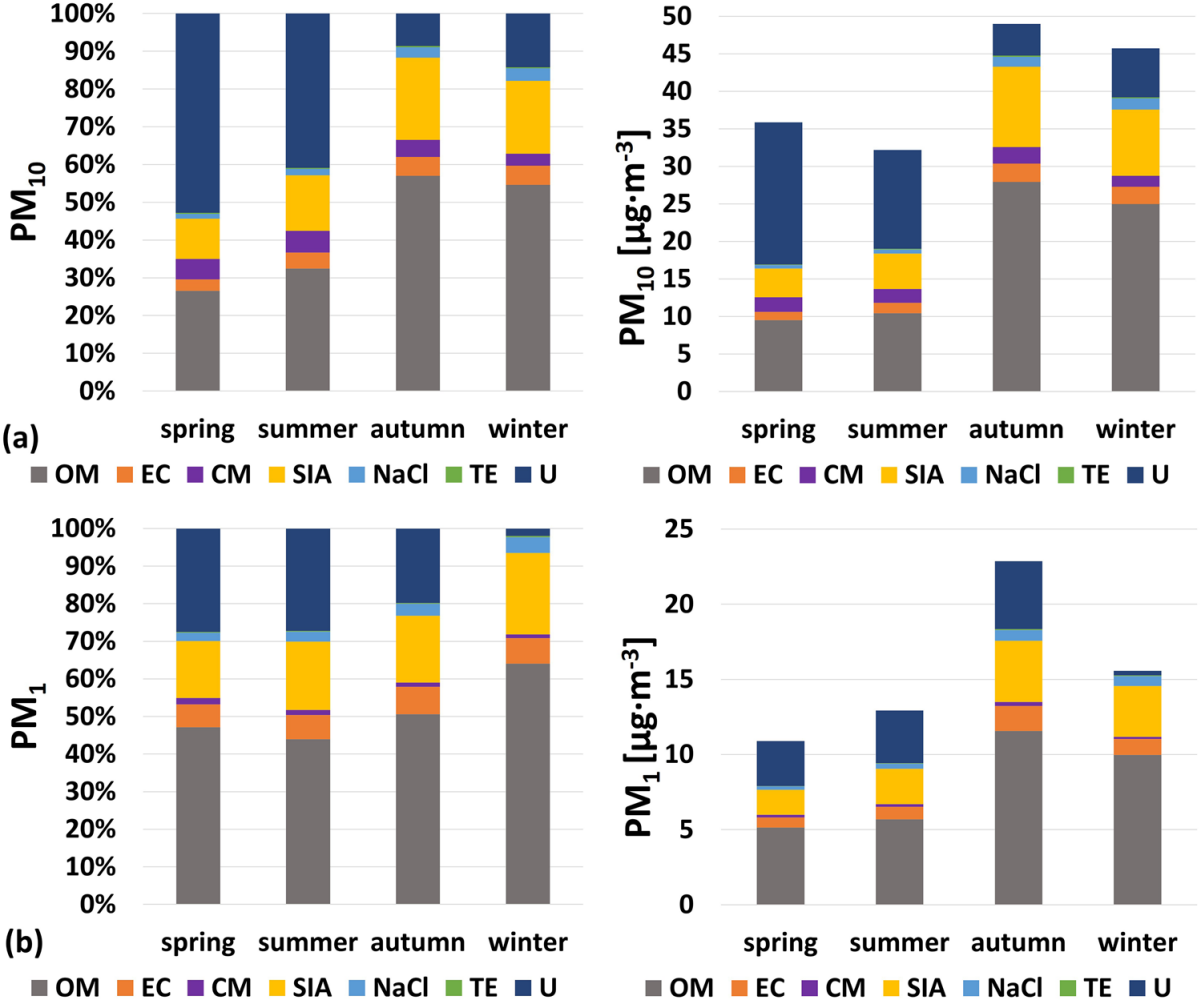
(**a**, **b**) Chemical mass closure results of PM_10_ and PM_1_ fractions for 4 different seasons, expressed in concentration units and percentages by weight (OM—organic matter, EC—elemental carbon, CM—soil/crustal matter, SIA—secondary inorganic ions, NaCl—salt, TE—trace elements, U—unidentified matter).

The seasonal attribution of sources of the analysed PM_1_ and PM_10_ fractions, based on the chemical mass closure approach, is presented in Fig. 6a,b.

Organic matter constituted the largest share of all the components of the analysed samples of particulate matter. In PM_10_ it was in the range from 27% (spring) to 57% (autumn), while in PM_1_ from 44% (summer) to 64% (winter). Furthermore, the method showed seasonal variability of elemental carbon concentration: an increase from 3.1% in spring to 5.1% in winter for PM_10_ and from 6.1% in spring to 6.8% in winter for PM_1_, with the highest percentage of EC for this fraction recorded in autumn (7.3%). For SIA, an increase was observed from 11% in spring to 19% in winter for PM_10_, with the highest share of SIA (22%) occurring in autumn. On the other hand, SIA within the PM_1_ fraction increased from 15% in spring to 22% in winter. A noticeable increase in salt concentration (NaCl) was found during winter. In the case of PM_10_, the total concentration in the warm months (spring + summer) was 10 μg m^−3^, while during the cold period (autumn + winter) it raised to 28 μg m^−3^. In the case of PM_1_ the seasonal differences are correspondingly smaller, the total NaCl concentration amounts to 6.1 μg m^−3^ (warm months) and 13 μg m^−3^ (cold months). The elevated concentrations of NaCl during cold season can be linked to the use of road salt to prevent icing on the streets. The percentage of trace elements varies slightly, depending on the fraction, from 0.2 to 0.4%. On the other hand, the percentage of crustal matter is higher in the warm season (spring + summer), compared to the cold season (autumn + winter). In the case of PM_1_, it is on average 2.1% (spring + summer) and 1.6% (autumn + winter), and in the case of PM_10_: 6.2% and 4.5%, respectively. This is probably related to soil resuspension processes active during spring and summer. Unidentified matter constituted from 1.5 to 27% in case of PM_1_, and from 8.0 to 52% in case of PM_10_. It is noticeable that for both fractions the biggest share of unidentified matter was observed during warm period (spring and summer).

## Conclusions

This is the first 1-year study dedicated to the fine (PM_1_) and coarse (PM_10_) particulate matter, collected simultaneously from April 21, 2018 to March 19, 2019 in Krakow, southern Poland. The chemical analyses carried out (incl. ion chromatography (IC), anion exchange chromatography with pulsed amperometric detection (HPAE-PAD), thermal-optical OC/EC method, energy dispersive X-ray fluorescence (EDXRF) and gas chromatography-mass spectrometry (GC/MS) allowed to explore the chemical characteristics of both PM fractions. Other authors also tried to analyse these two fractions (PM_1_ and PM_10_) of particulate matter in the urban environment^75–78^. However, the number of analytical methods used was smaller. In addition, this is the first time when PM_1_ fraction was used to provide isotope mass-balance in Poland. Application of the mass closure method, based on performed analyses, allowed to obtain seasonal variability of the chemical composition of two particulate matter fractions. The dominant revealed component was the organic matter (from 27 to 64% depending on the season of the year and the analysed fraction). The results from HPAE-PAD and GC/MS demonstrated that carbohydrates and PAHs had no significant share in organic carbon reservoir. The HPAE-PAD technique showed high concentrations of levoglucosan (marker of biomass combustion) during the heating-season. Its average concentrations for the entire measurement period were 0.16 µg·m^−3^ and 0.33 µg·m^−3^ for PM_1_ and PM_10_ fractions, respectively. Both of analyses (HPAE-PAD and GC/MS) provided valuable data on the presence of sugars and PAHs in the samples. In this work, we focused on the analysis of the carbonaceous fraction, but further work on the obtained data is highly recommended and planned in the future. They may provide valuable information in aspects other than those discussed in this work (e.g. the use of levoglucosan as a marker of biomass combustion using other data processing methods, or health aspects in the case of PAHs). The application of the carbon isotope-mass balance method to identify the sources of PM_1_ and PM_10_ fractions of suspended particulate matter emissions allowed to distinguish three main emission sources: (1) the emissions from coal combustion, (2) the emissions related to road transport, and (3) the biogenic emissions. The results presented here clearly show that during the heating season emissions from coal combustion had the biggest contribution to the PM_10_ carbonaceous aerosol reservoir (44%), and together with the biogenic emissions they were the biggest contributors to PM_1_ (41% and 44%, respectively). In the non-heating season, the dominant source of carbon were biogenic emissions (48% of PM_10_, 54% of PM_1_). Since the research was conducted just before the Krakow City Council introduced a total ban on combustion of solid fuels (starting from September 1, 2019), it would be recommended to conduct similar studies in the coming years to check whether the introduced regulations resulted in any noticeable improvement in air quality in Krakow.

### Supplementary Information

Supplementary Information.

## Data Availability

The datasets used and/or analysed during the current study available from the corresponding author on reasonable request.
