# Supplementary material for: Source attribution of carbonaceous fraction of particulate matter in the urban atmosphere based on chemical and carbon isotope composition
Source: Sci Rep. 2024 Mar 27;14:7234. doi: 10.1038/s41598-024-57829-x (PMC11366020; doi:10.1038/s41598-024-57829-x)
Supplement: Supplementary file 1 — Supplementary Information. [file 41598_2024_57829_MOESM1_ESM.pdf]

Supplementary Information

Source attribution of carbonaceous fraction of particulate matter in the urban atmosphere based on chemical and carbon isotope composition

Alicja Skiba[1], Katarzyna Styszko[2*], Anna Tobler[3,4], Roberto Casotto[3], Zbigniew Gorczyca[1], Przemysław Furman[1], Lucyna Samek[1], Dariusz Wideł[5], Mirosław Zimnoch[1], Anne Kasper-Giebl[6], Jay G. Slowik[3], Kaspar R. Daellenbach[3], Andre S. H. Prevot[3], Kazimierz Różański[1]

[1] AGH University of Krakow, Faculty of Physics and Applied Computer Science, Krakow, Poland
[2] AGH University of Krakow, Faculty of Energy and Fuels, Krakow, Poland
[3] Laboratory of Atmospheric Chemistry, Paul Scherrer Institute, 5232 Villigen-PSI, Switzerland
[4] Datalystica Ltd., Park innovAARE, 5234 Villigen, Switzerland
[5] Jan Kochanowski University, Institute of Chemistry, Uniwersytecka 7 Street, 25-406 Kielce, Poland
[6] Institute for Chemical Technologies and Analytics, TU-Wien, 1060 Vienna, Austria
*Corresponding author: styszko@agh.edu.pl

A. Filters collection details

The filters were conditioned prior to collection. They were baked at 450 °C for 4 h and then transferred to a desiccator filled with water for 2 h. Then, they were conditioned in a thermostated chamber (temperature 20± 1° C and relative humidity 50±5 %) for 48 h. The preparation procedure was completed by weighing the filters with an OHAUS Discovery DV215CD balance (precision 0.01 mg). The weight of the deposited fraction of the particulate matter was defined as a difference in filter weights before and after the collection of the given fraction.

B. Isotope analysis aggregation periods and TC weight average

Table 1S. Samples of particulate matter subjected to carbon isotope analysis and their assignment to the seasons supplemented with the corresponding weighted averages of the total carbon present in each sample.

Season	Particulate matter fraction		Dates of the samples subject to aggregation and their total number	TC (weighted average) [mg]	
	PM_1	PM_{10}		PM_1	PM_{10}
non-heating	April 2018	April 2018	Every sample (21.04.2018-30.04.2018) Σ10	3.3	5.33
	May 2018	-	Every fourth sample (03.05.2018-31.05.2018) Σ8	2.65	-
	June 2018	-	Every fourth sample (04.06.2018-28.06.2018) Σ7	2.77	-
	July 2018	July 2018	Every fourth sample (02.07.2018-30.07.2018) Σ8	2.72	4.85
	-	August 2018	Every fourth sample (03.08.2018-31.08.2018) Σ8	-	5.17
heating	October 2018	October 2018	Every sample (23.10.2018-31.10.2018) Σ9	2.94	5.06
	November 2018	November 2018	Every fourth sample (03.11.2018-27.11.2018) Σ7	7.66	17.03
	December 2018	December 2018	Every fourth sample (01.12.2018-29.12.2018) Σ8	9.23	20.26

	January 2019	January 2019	Every fourth sample (02.01.2019-30.01.2019) Σ8	6.86	18.81
	February 2019	February 2019	Every fourth sample (03.02.2019-27.02.2019) Σ7	8.28	21.93
	March 2019	March 2019	Every fourth sample (03.03.2019-19.03.2019) Σ5	4.54	9.5

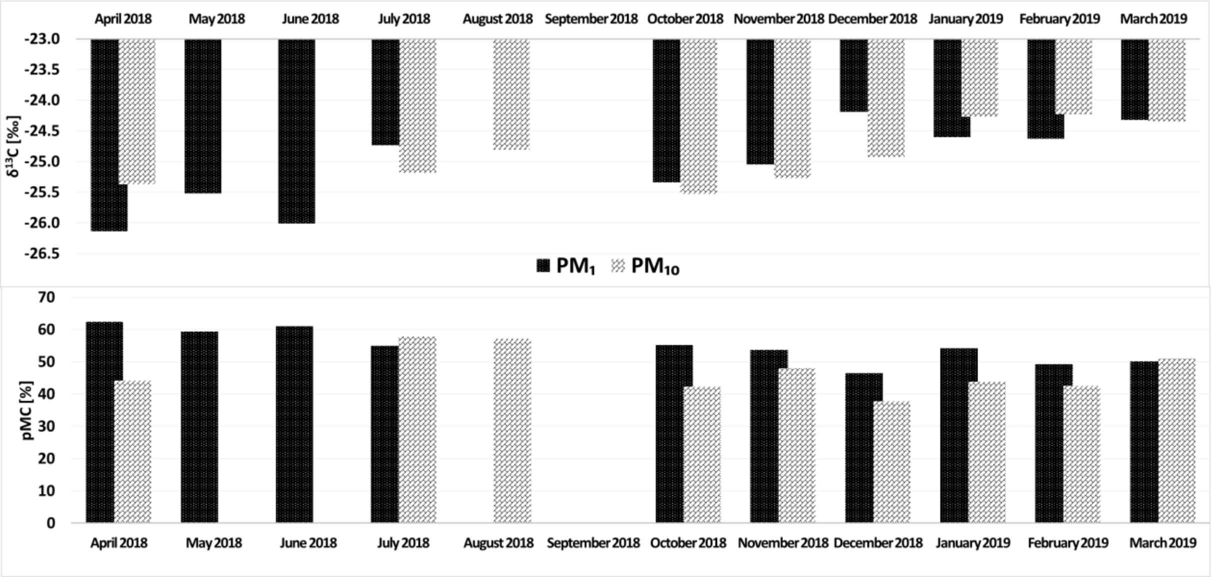

Fig. 1S. Results of analyses of the isotopic composition of carbon ($\delta^{13}C_{TC}$ and pMC_{TC}) in aggregated monthly samples of PM_1 and PM_{10} fractions.

C. Methods details

I. OC/EC measurements with thermal-optical method using Sunset Laboratory OCEC Lab Aerosol Analyzer (USA) details (EUSAAR2 protocol)

The samples were exposed to different thermal conditions, thus allowing carbonaceous material to be thermally desorbed from the filter punch in a helium atmosphere (temperature: 200 - 650 °C), followed by an oxidizing atmosphere (oxygen/helium mixture, temperature: 500 - 850 °C) [1]. The accuracy and repeatability of the analyses were controlled through regular measurements of a sucrose solution containing 50 µg of carbon in 10 µl of the solution or by using reference filters. The detection limits for OC and EC were equal to 0.54 $\mu g\cdot m^{-3}$ and 0.09 $\mu g\cdot m^{-3}$, respectively.

II. Inorganic ions analysis details

Inorganic anions (F^-, Cl^-, NO_2^-, Br^-, NO_3^-, PO_4^{3-}, SO_4^{2-}) and cations (Na^+, K^+, Mg^{2+}, Ca^{2+}, NH_4^+) concentrations were analysed with isocratic ion chromatography (IC) as follows: two circular punches (ø 10 mm) per filter were extracted under ultrasonic agitation for 40 min, in either 1.5 ml of Milli-Q water (anions) or in 1.5 ml of the 12 mM methanesulfonic acid (MSA) (cations). In the next step, the extracts were centrifuged (4000 rpm for 5 min) and then transferred to chromatographic vials. Ion chromatography analysis was performed with ICS-1100 instrument (Thermo Scientific™). Electrochemical suppression with AERS 500-4 mm and CERS 500-4 mm suppressors for anions and cations, respectively have been made. After suppression quantification was made with conductivity detector. Calibration was performed against external standards diluted from stock solutions (Thermo

Scientific™). The limit of detection calculation method and further equipment details are described in [4].

III. Polycyclic aromatic hydrocarbons analysis details and results

Sixteen polycyclic aromatic hydrocarbons (acenaphthene (Acn), acenaphthylene (Acy), anthracene (Ant), benzo[b]fluoranthene (BbF), benzo[a]anthracene (BaA), benzo[a]pyrene (BaP), benzo[ghi]perylene (BghiP), benzo[k]fluoranthene (BkF), chrysene (Chry), dibenzo[ah]anthracene (DahA), fluoranthene (Flt), fluorene (Flu), indeno[1,2,3-cd]pyrene (IP), naphthalene (Nap), phenanthrene (Phen) and pyrene (Pyr)) were analysed with gas chromatography coupled with mass spectrometry system (GC/MS). Model Clarus 600/600T from Perkin Elmer (USA) was used. Polycyclic aromatic hydrocarbons were separated on the Elite-5MS (30 m × 0.25 mm × 0.25 µm) capillary column. Altogether 48 aggregated samples (2, 3 or 4 days – depending on the sampling month, (cf. Table 2S Supplementary Information) were analysed for PM_1 and PM_{10} fractions (24 samples for each particulate matter fraction).

The circular punches of individual filters (ø 18 mm) were combined and extracted twice with 3 ml of dichloromethane and 2 ml of cyclohexane for 40 min at 50 rpm. The volume of combined extracts was reduced to 500 µl in a thermoblock (AccuBlock Digital Dry Bath Labnet, Woodbridge, USA), using gentle stream of argon at 35 °C. The extracts were then centrifuged (12.000 rpm). The final concentrates (200 µl) were transferred into chromatographic vials and analysed with GC/MS. The procedure was described in detail in [5] and was slightly modified in order to analyse polycyclic aromatic hydrocarbons. In this work the flow of helium carrier gas (99.9999 % purity) through a GC column was set at 1 $ml \cdot min^{-1}$, while volume of individual injections was 1 µl. The temperature program for PAHs analysis was as follows: 60 °C for 1 min, from 60 °C to 300 °C at 15 °C min^{-1} and finally 13 min at 300 °C. The standards of known PAHs concentration (EPA 525 PAH Mix A, 2 ml, 10 000 $ng \cdot ml^{-1}$ (Sigma Aldrich) were used for the calibration. The retention times, characteristic ions of tested analytes and validation parameters of the method are presented in Table 3S (Supplementary Information).

Table. 2S PAHs identified in PM_{10} and PM_1 fractions, followed by each sample aggregation dates and percentage share of carbon from PAHs in the organic carbon reservoir.

Name	Date	PM_{10}			PM_1		
		Determined PAHs	Total no. of determined PAHs	Share of carbon from PAHs in OC [%]	Determined PAHs	Total no. of determined PAHs	Share of carbon from PAHs in OC [%]
Apr_2018	21.04.2018<br>25.04.2018<br>29.04.2018	Phen, Flt, Pyr, BaA, Chry, BbF, BkF, BaP, IP, BghiP	10	0.008	BbF	1	0.001
May_1	03.05.2018<br>07.05.2018<br>11.05.2018	Phen, Flt, Pyr, BaA, Chry, BbF, BkF, BaP, IP, BghiP	10	0.007	-	0	-
May_2	15.05.2018<br>19.05.2018	Flt, Pyr, Chry, BbF	4	0.003	BbF	1	0.001
May_3	23.05.2018<br>27.05.2018	Flt, Pyr, BaA, Chry, BbF,	8	0.004	BbF	1	0.001

	31.05.2018	BkF, BaP, BghiP					
Jun_1	04.06.2017<br>08.06.2018<br>12.06.2018	Phen, Flt, Pyr, BaA, Chry, BbF, BkF, BaP	8	0.007	BbF	1	0.001
Jun_2	16.06.2018<br>20.06.2018<br>24.06.2018<br>28.06.2018	Flt, Pyr, Chry, BbF, BaP	5	0.001	BbF	1	0.001
Jul_1	02.07.2018<br>06.07.2018<br>09.07.2018<br>14.07.2018	Phen, Flt, Pyr, BaA, Chry, BbF, BkF, BaP, IP, BghiP	10	0.015	Flt, Pyr, BbF	3	0.001
Jul_2	18.07.2018<br>22.07.2018<br>26.07.2018<br>30.07.2018	Flt, Pyr, Chry, BbF, BaP	5	0.001	BbF	1	0.001
Aug_1	03.08.2018<br>07.08.2018<br>11.08.2018<br>15.08.2018	Flt, Pyr, BaA, Chry, BbF, BkF, BaP, IP, BghiP	9	0.006	Flt, Pyr, BbF	3	0.002
Aug_2	19.08.2018<br>23.08.2018<br>27.08.2018<br>31.08.2018	Phen, Flt, Pyr, BaA, Chry, BbF, BkF, BaP, IP, BghiP	10	0.015	Pyr, BbF	2	0.001
Sep_1	04.09.2018<br>08.09.2018<br>12.09.2018<br>16.09.2018	Phen, Flt, Pyr, BaA, Chry, BbF, BkF, BaP, IP, BghiP	10	0.004	BbF	1	0.001
Sep_2	20.09.2018<br>24.09.2018<br>27.09.2018	Flt, Pyr, BaA, Chry, BbF, BkF, BaP, BghiP	8	0.004	Flt, BbF	2	0.003
Oct	26.10.2018<br>30.10.2018	Phen, Flt, Pyr, BaA, Chry, BbF, BkF, BaP, IP, BghiP	10	0.015	Phen, Flt, Pyr, BaA, Chry, BbF, BkF, BaP, IP, BghiP	10	0.04
Nov_1	03.11.2018<br>07.11.2018<br>11.11.2018	Phen, Ant, Flt, Pyr, BaA, Chry, BbF, BkF, BaP, IP, DahA, BghiP	12	0.055	Phen, Flt, Pyr, BaA, Chry, BbF, BkF, BaP, IP, DahA, BghiP	11	0.08
Nov_2	15.11.2018<br>19.11.2018	Phen, Ant, Flt, Pyr, BaA,	12	0.07	Phen, Flt, Pyr, BaA,	11	0.05

	23.11.2018<br>27.11.2018	Chry, BbF, BkF, BaP, IP, DahA, BghiP			Chry, BbF, BkF, BaP, IP, DahA, BghiP		
Dec_1	01.12.2018<br>05.12.2018<br>09.12.2018<br>13.12.2018	Acy, Flu, Phen, Ant, Flt, Pyr, BaA, Chry, BbF, BkF, BaP, IP, DahA, BghiP	14	0.07	Phen, Ant, Flt, Pyr, BaA, Chry, BbF, BkF, BaP, IP, DahA, BghiP	12	0.08
Dec_2	17.12.2018<br>21.12.2018<br>25.12.2018<br>29.12.2018	Nap, Acy, Flu, Phen, Ant, Flt, Pyr, BaA, Chry, BbF, BkF, BaP, IP, DahA, BghiP	15	0.09	Nap, Acy, Phen, Ant, Flt, Pyr, BaA, Chry, BbF, BkF, BaP, IP, DahA, BghiP	14	0.09
Jan_1	02.01.2019<br>06.01.2019<br>10.01.2019<br>14.01.2019	Phen, Ant, Flt, Pyr, BaA, Chry, BbF, BkF, BaP, IP, DahA, BghiP	12	0.06	Phen, Ant, Flt, Pyr, BaA, Chry, BbF, BkF, BaP, IP, BghiP	11	0.05
Jan_2	18.01.2019<br>22.01.2019<br>26.01.2019<br>30.01.2019	Acy, Flu, Phen, Ant, Flt, Pyr, BaA, Chry, BbF, BkF, BaP, IP, DahA, BghiP	14	0.09	Nap, Phen, Ant, Flt, Pyr, BaA, Chry, BbF, BkF, BaP, IP, DahA, BghiP	13	0.06
Feb_1	03.02.2019<br>07.02.2019<br>11.02.2019<br>15.02.2019	Nap, Acy, Flu, Phen, Ant, Flt, Pyr, BaA, Chry, BbF, BkF, BaP, IP, DahA, BghiP	15	0.09	Phen, Ant, Flt, Pyr, BaA, Chry, BbF, BkF, BaP, IP, DahA, BghiP	12	0.09
Feb_2	19.02.2019<br>23.02.2019<br>27.02.2019	Nap, Acy, Flu, Phen, Ant, Flt, Pyr, BaA, Chry, BbF, BkF, BaP, IP, DahA, BghiP	15	0.21	Phen, Ant, Flt, Pyr, BaA, Chry, BbF, BkF, BaP, IP, DahA, BghiP	12	0.08
Mar_1	03.03.2019<br>07.03.2019<br>11.03.2019<br>15.03.2019	Nap, Acy, Phen, Ant, Flt, Pyr, BaA, Chry, BbF, BkF, BaP, IP, DahA, BghiP	14	0.09	Phen, Flt, Pyr, BaA, Chry, BbF, BkF, BaP, IP, BghiP	10	0.04

The retention times, characteristic ions of tested analytes and validation parameters of the method are shown in Table 3S. Data quality was determined based on the limits of detection (LOD), limits of quantification (LOQ), and the linearity of the calibration line that was estimated by analysing the 11-points calibration curves (2.5–5000 ng·ml^{-1}). The calibration curves of all the polycyclic aromatic hydrocarbons were highly linear ($0.991 < R^2 < 0.999$). For further quality assurance, known amount of mixed standards were spiked on blank filters and analysed in the same steps as samples. Blank samples analysis showed none of the PAHs of interest. The percent recoveries for all the target compounds were in the range of 88–97 %.

Table 3S. Chromatographic and mass spectrometric characterization of target analytes: retention time, mass of characteristic ions, correlation coefficients-R^2 and LOQ.

Compound	Retention time [min]	Precursor-products ions $m\ z^{-1}$	R^2	LOQ [$ng \cdot ml^{-1}$]
naphthalene	6.11	128	0.991	1.73
acenaphthylene	8.45	152	0.994	3.27
acenaphthene	8.77	153	0.996	2.16
fluorene	9.72	166	0.998	1.90
phenanthrene	11.68	178	0.996	13.77
anthracene	11.79	178	0.997	4.51
fluoranthene	14.32	202	0.992	4.01
pyrene	14.82	202	0.994	0.84
benzo[a]anthracene	17.60	228	0.997	0.38
chrysene	17.69	228	0.999	0.63
benzo[b]fluoranthene	19.95	252	0.991	1.53
benzo[k]fluoranthene	20.01	252	0.995	0.01
benzo[a]pyrene	20.6	252	0.995	0.86
indeno[1,2,3-cd]pyrene	22.98	276	0.998	1.70
dibenzo[ah]anthracene	23.06	278	0.999	5.61
benzo[ghi]perylene	23.64	276	0.996	0.76

IV. Elemental composition analysis details

The elemental analysis of the filter material comprised 17 elements: Cl, K, Ca, Ti, V, Cr, Mn, Fe, Co, Ni, Zn, Cu, Br, Rb, Sr, As and Pb. The concentrations of the measured elements were quantified by the energy dispersive X-ray fluorescence (EDXRF) method. In brief, the measurements were carried out under the following conditions: voltage of 55 kV, current of 30 mA, measuring time of 2400 s. The EDXRF spectrometer was calibrated using thin film standards (Micromatter, Washington, USA). The calibration was verified by the analysis of U.S. NIST standard SRM 2783 (Air Particulate Matter on Filter Media). Detection limits and detailed description of the method is presented elsewhere[2,3].

D. Mass closure method assumptions

Table 4S. The categories adopted in the chemical mass closure method.

Categories	Abbrev.	Equation	References
**Secondary inorganic ions**	SIA	$SIA = [NH_4^+] + [SO_4^{2-}] + [NO_3^-]$	[11–13]
**Organic matter**	OM	$OM = 1.8 \times [OC]$	[14,15]
**Elemental carbon**	EC	$EC = [EC]$	[11–13,16,17]
**Crustal matter**	CM	$CM^* = 1.63 \times [Ca] + 1.94 \times [Ti] + 2.42 \times [Fe] + 2.4 \times [K]$	[13,18]
**Salt**	NaCl	$NaCl = 2.54 \times [Na^+]$	[19]
**Trace elements**	TE	$TE = [V] + [Cr] + [Mn] + [Co] + [Ni] + [Cu] + [Zn] + [As] + [Br] + [Rb] + [Sr] + [Pb]$	[13,16]
**Unidentified matter**	U	$U = [PM_x] - [\Sigma_{identified\ components\ of PM_x}]$	[14,16]

* The original equation from[18] has the form: $\boldsymbol{CM} = \mathbf{2.2} \times [\boldsymbol{Al}] + \mathbf{2.49} \times [\boldsymbol{Si}] + \mathbf{1.63} \times [\boldsymbol{Ca}] + \mathbf{1.94} \times [\boldsymbol{Ti}] + \mathbf{2.42} \times [\boldsymbol{Fe}]$. However, the elements Al and Si were not measured in the EDXRF analyses performed in this study. The equation was supplemented after[13] with 2.4 × [K] due to stoichiometric concentration of K_2O.

E. Summary of PAHs and carbohydrates results

Table 5S. Summary of the results obtained for polycyclic aromatic hydrocarbons and carbohydrates analyses for PM_1 and PM_{10} fractions, followed by number of samples >LOQ.

**PAHs**	**PM_1**				**PM_{10}**			
**Concentration [ng·m^{-3}]**	min	max	average	samples >LOQ	min	max	average	samples >LOQ
Naphthalene	0.03	0.05	0.03	2	0.02	0.06	0.03	4
Acenaphthylene	0.04	0.04	0.04	1	0.04	0.17	0.10	7
Acenaphthene	<LOQ	<LOQ	<LOQ	0	<LOQ	<LOQ	<LOQ	0
Fluorene	<LOQ	<LOQ	<LOQ	0	0.03	0.41	0.23	5
Phenathrene	0.20	1.67	0.71	10	0.16	7.44	2.23	16
Anthracene	0.06	0.45	0.18	6	0.17	3.10	1.33	8
Fluoranthene	0.05	4.85	1.77	13	0.07	17.64	3.57	22
Pyrene	0.01	4.38	1.62	13	0.02	15.31	3.05	22
Benzo[a]anthracene	0.19	3.13	1.52	10	0.01	11.55	2.35	19
Chrysene	0.19	3.49	1.87	8	0.01	12.62	2.33	22
Benzo[b]fluoranthene	0.05	5.35	1.49	21	0.09	21.94	4.24	22
Benzo[k]fluoranthene	0.05	1.72	1.04	10	0.00	7.97	1.64	19

Benzo[a]pyrene	0.20	2.70	1.46	10	0.01	11.01	2.15	21
Indeno[1,2,3-cd]pyrene	0.05	2.09	1.16	10	0.00	10.40	2.15	18
Dibenz[a,h]anthracene	0.07	0.52	0.26	7	0.13	2.71	1.15	9
Benzo[g,h,i]perylene	0.05	1.90	1.09	10	0.01	9.54	2.11	18
**Carbohydrates**	**PM_1**				**PM_{10}**			
**Concentration [$\mu g \cdot m^{-3}$]**	min	max	average	days >LOQ	min	max	average	days >LOQ
Inositol	0.043	0.051	0.05	5	0.043	0.11	0.07	8
Erythritol	<LOQ	<LOQ	<LOQ	0	0	<LOQ	<LOQ	<LOQ
Xylitol	<LOQ	<LOQ	<LOQ	0	0	<LOQ	<LOQ	<LOQ
Levoglucosan	0.024	0.901	0.16	74	0.029	1.95	0.33	78
Arabitol	<LOQ	<LOQ	<LOQ	0	0.015	0.15	0.04	46
Mannosan	0.038	0.125	0.07	14	0.037	0.23	0.09	27
Trehalose	<LOQ	<LOQ	<LOQ	0	0.09	0.36	0.2	4
Mannitol	<LOQ	<LOQ	<LOQ	0	0.086	0.1	0.09	3
Galactosan	0.025	0.029	0.03	3	0.023	0.1	0.05	23
Glucose	0.028	0.028	0.03	1	0.024	0.09	0.05	41
Galactose	<LOQ	<LOQ	<LOQ	0	0	<LOQ	<LOQ	<LOQ
Fructose	<LOQ	<LOQ	<LOQ	0	0	<LOQ	<LOQ	<LOQ
Cellobiose	<LOQ	<LOQ	<LOQ	0	0.185	0.19	0.19	1
Sucrose	<LOQ	<LOQ	<LOQ	0	0	<LOQ	<LOQ	<LOQ

Table 6S. Comparison of average concentrations of PAHs obtained in this study with the results of other research groups, obtained for different locations.

**City, country**	**Fraction**	**Period**	**ΣPAHs [$ng \cdot m^{-3}$]**	**Reference**
Krakow, Poland	PM_1	April 2018 - March2019	0.96	this study
	PM_{10}		1.98	
	PM_{10}	winter 2014	8.47	[6]
Kaunas, Lithuania	$PM_{2.5}$	winter 2009	36.5	[7]
	$PM_{2.5}$	spring 2009	23.6	
Thessaloniki, Greece	PM_{10}	July 1997 - July 1998	26	[8]
Oporto, Portugal	$PM_{2.5}$	winter 2013/14	16.3	[9]
	$PM_{2.5}$	summer 2014	5.6	

Florence, Italy	$PM_{2.5}$	winter 2013/14	7.75	
	$PM_{2.5}$	summer 2014	3.02	
Athens, Greece	$PM_{2.5}$	winter 2013/14	3.44	
	$PM_{2.5}$	summer 2014	0.658	
Canoas, Brazil	PM_1	August 2011 - July 2013	1.52	10
Sapucaia do Sul, Brazil	PM_1	August 2011 - July 2013	1.99	

F. Isotope-mass balance method assumptions

Table 7S. The data used in isotope-mass balance equations for heating and non-heating season for aggregated samples of PM_1 and PM_{10}.

**Season**	**$\delta^{13}C_{bio}$ [‰]**	**$\delta^{13}C_{coal}$ [‰]**	**$\delta^{13}C_{traff}$ [‰]**	**pMC_{bio} [%]**	**pMC_{traff} [%]**	**pMC_{coal} [%]**
**non-heating**	-25.0	-23.3	-27.6	105	10	0
**heating**	-25.0	-23.3	-27.6	115	10	0

G. The sensitivity analysis of uncertainties involved in isotope-mass balance approach

a)

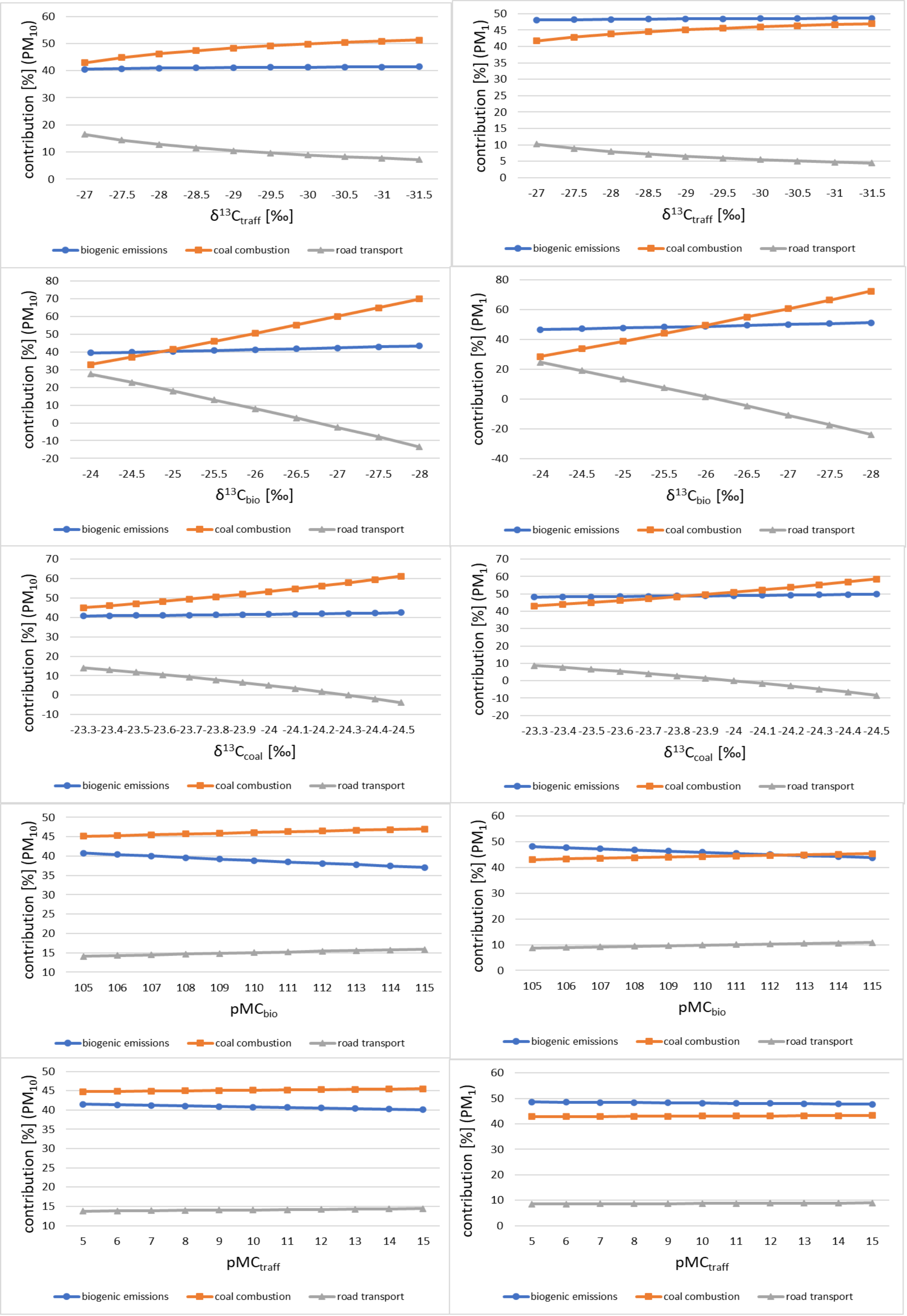

b)

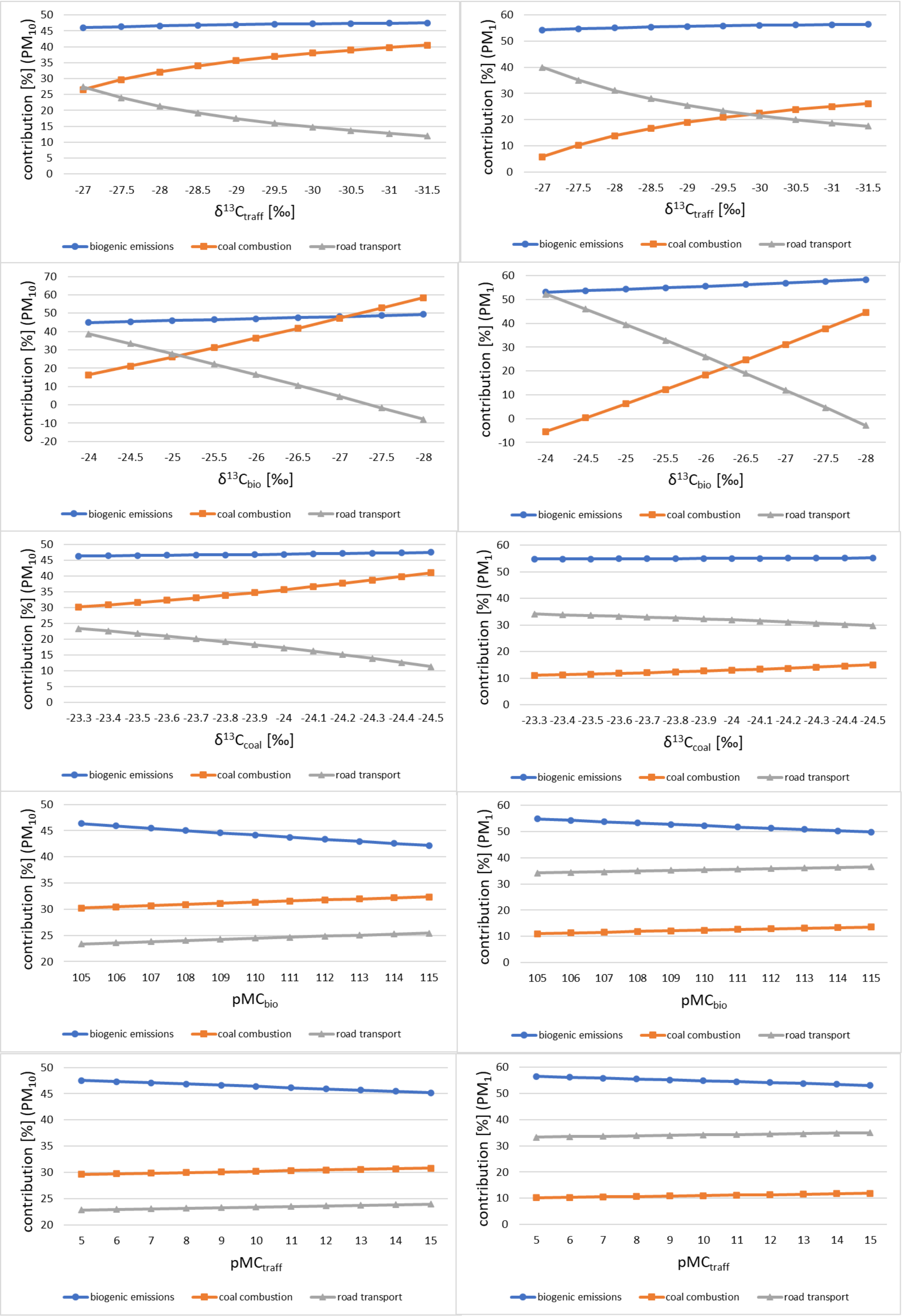

Fig. 2S. The sensitivity analysis of source apportionment calculations applied separately for the heating (a) and non-heating (b) season.

References

1. Cavalli, F., Viana, M., Yttri, K. E., Genberg, J. & Putaud, J. P. Toward a standardised thermal-optical protocol for measuring atmospheric organic and elemental carbon: The EUSAAR protocol. *Atmospheric Measurement Techniques* **3**, 79–89 (2010).

2. Samek, L., Stegowski, Z., Styszko, K., Furman, L. & Fiedor, J. Seasonal contribution of assessed sources to submicron and fine particulate matter in a Central European urban area. *Environmental Pollution* **241**, 406–411 (2018).

3. Samek, L. *et al.* Seasonal variations of chemical composition of PM2.5 fraction in the urban area of Krakow, Poland: PMF source attribution. *Air Quality, Atmosphere and Health* 89–96 (2020). doi:10.1007/s11869-019-00773-x

4. Samek, L. *et al.* Quantitative Assessment of PM2.5 Sources and Their Seasonal Variation in Krakow. *Water, Air, and Soil Pollution* **228**, (2017).

5. Wideł, D., Jedynak, K. & Witkiewicz, Z. Application of ordered micro-mesoporous carbon materials activated by steam and CO2 or KOH in solid-phase extraction of selected phthalates from aqueous samples. *Desalination and Water Treatment* **232**, 91–104 (2021).

6. Styszko, K. *et al.* Polycyclic aromatic hydrocarbons and their nitrated derivatives associated with PM10 from Krakow city during heating season. *E3S Web of Conferences* **10**, (2016).

7. Kliucininkas, L. *et al.* Indoor and outdoor concentrations of fine particles, particle-bound PAHs and volatile organic compounds in Kaunas, Lithuania. *Journal of Environmental Monitoring* **13**, 182–191 (2011).

8. Manoli, E., Kouras, A. & Samara, C. Profile analysis of ambient and source emitted particle-bound polycyclic aromatic hydrocarbons from three sites in northern Greece. *Chemosphere* **56**, 867–878 (2004).

9. Alves, C. A. *et al.* Polycyclic aromatic hydrocarbons and their derivatives (nitro-PAHs, oxygenated PAHs , and azaarenes) in PM 2.5 from Southern European cities. *Science of the Total Environment* **595**, 494–504 (2017).

10. Agudelo-Castañeda, D. M. & Teixeira, E. C. Seasonal changes, identification and source apportionment of PAH in PM1.0. *Atmospheric Environment* **96**, 186–200 (2014).

11. Maenhaut, W., Schwarz, J., Cafmeyer, J. & Chi, X. Aerosol chemical mass closure during the EUROTRAC-2 AEROSOL Intercomparison 2000. *Nuclear Instruments and Methods in Physics Research, Section B: Beam Interactions with Materials and Atoms* **189**, 233–237 (2002).

12. Andrews, E. *et al.* Concentration and composition of atmospheric aerosols from the 1995 SEAVS experiment and a review of the closure between chemical and gravimetric measurements. *Journal of the Air and Waste Management Association* **50**, 648–664 (2000).

13. Rogula-Kozłowska, W., Klejnowski, K., Rogula-Kopiec, P., Mathews, B. & Szopa, S. A Study on the Seasonal Mass Closure of Ambient Fine and Coarse Dusts in Zabrze, Poland. *Bulletin of Environmental Contamination and Toxicology* **88**, 722–729 (2012).

14. Hand, J. L. Spatial and Seasonal Patterns and Temporal Variability of Haze and its Constituents in the United States. *IMPROVE Report V* (2011).

15. Simon, H., Bhave, P. V., Swall, J. L., Frank, N. H. & Malm, W. C. Determining the spatial and seasonal variability in OM/OC ratios across the US using multiple regression. *Atmospheric Chemistry and Physics* **11**, 2933–2949 (2011).

16. Sillanpää, M. *et al.* Chemical composition and mass closure of particulate matter at six urban sites in Europe. *Atmospheric Environment* **40**, 212–223 (2006).

17. Furman, P. *et al.* Seasonal variability of PM10 chemical composition including 1,3,5-triphenylbenzene, marker of plastic combustion and toxicity in wadowice, south poland. *Aerosol and Air Quality Research* **21**, 1–12 (2021).

18. Malm, W. C., Sisler, J. F., Huffman, D., Eldred, R. A. & Cahill, T. A. *Spatial and seasonal trends in particle concentration and optical extinction in the United States*. *JOURNAL OF GEOPHYSICAL RESEARCH* **99**, (1994).

19. Chow, J. C., Lowenthal, D. H., Chen, L. W. A., Wang, X. & Watson, J. G. Mass reconstruction methods for PM2.5: a review. *Air Quality, Atmosphere and Health* **8**, 243–263 (2015).
